# A study on the neurodevelopment outcomes of late preterm infants

**DOI:** 10.1186/s12883-019-1336-0

**Published:** 2019-05-30

**Authors:** Jia You, Bilal Haider Shamsi, Mei-chen Hao, Chun-Hong Cao, Wu-Yue Yang

**Affiliations:** 1 Department of Child Health Care, Xi’an Maternal and Child Health Care Hospital, NO 73, Street Xidajie, Xi’an, 710003 Shaanxi China; 2 Department of Public Health Care, Xi’an Maternal and Child Health Care Hospital, Xi’an, 710003 Shaanxi China; 3 Pediatrics Department, Shenmu Hospital, Shenmu, 719300 Shaanxi China; 4 Division of Neurobiology and Physiology, Department of Basic Medical Sciences, School of Medicine, Zhejiang University , Hangzhou, 310000 Zhejiang China

**Keywords:** Late preterm infants, Motor disorders, Autism spectrum disorders

## Abstract

**Background:**

The study is intended to fill the knowledge gap about the neuropsychology and neuromotor developmental outcomes, and identify the perinatal risk factors for late preterm infants (LPIs 34~36 weeks GA) born with uncomplicated vaginal birth at the age of 24 to 30 months.

**Methods:**

The parents/guardians of 102 late preterm infants and 153 term infants, from 14 community health centers participated in this study. The Modified Checklist for Autism in Toddlers (M-CHAT) questionnaire, the Chinese version of Gesell Development Diagnosis Scale (GDDS), and the Sensory Integration Schedule (SIS), a neurological examination for motor disorders (MD) were carried out. Infants screening positive to the M-CHAT were referred to specialist autism clinics.

**Results:**

Forty-six LPIs (45.1%) scored low in GDDS. Nine LPIs (8.8%) scored positive on M-Chat. 8.8% of LPIs (9 out of 102) were diagnosed MD (*p* <  0.05). Compared with their full-term peers, LPIs had statistically lower scores in GDDS and the Child Sensory Integration Checklist. LPIs who had positive results on M-CHAT showed unbalanced abilities in every part of GDDS. Risk factors of twin pregnancies, pregnancy induced hypertension and premature rupture of membranes had negative correlation with GDDS (all *p* <  0.05). Birth weight and gestational age were positively correlated with GDDS.

**Conclusions:**

LPIs shall be given special attention as compared to normal deliveries, as they are at increased risk of neurodevelopment impairment, despite being born with no major problems. Some perinatal factors such as twin pregnancies, and pregnancy induced hypertension etc. have negative effects on their neurodevelopment. Regular neurodevelopmental follow- up and early intervention can benefit their long term outcomes.

**Electronic supplementary material:**

The online version of this article (10.1186/s12883-019-1336-0) contains supplementary material, which is available to authorized users.

## Background

Over the past 2 decades, incidence of late preterm infants (LPIs), defined as those born between 34 + 0 and 36 + 6 weeks of gestation, have become more prevalent [1]. According to the recent literature, LPIs account for approximately 84% of preterm births [2]. A survey of 80 hospitals in China in 2005 showed that 62.6% of preterm infants hospitalized in neonatal units were LPIs [3]. Social concerns have long focused on neurodevelopment of extremely preterm and extremely low birth-weight infants but LPIs are left unfocused. Many neuropsychological and behavior problems have been reported with these children such as autism spectrum disorders (ASD), attention deficit hyperactivity disorder (ADHD), anxiety, and depression [4–6], however neurodevelopment of LPIs has attracted less attention. LPIs, unlike extremely preterm infants, are usually assumed to grow healthily like normal full term infants, and the follow up checkups are focused only on the physical milestones hence the psychological and behavioral development is overlooked. In recent years, studies have looked into development of recognition, social development, emotional integrity, and behaviors etc. but systematical studies are not quantitative [7]. Findings with LPIs’ neurodevelopmental outcomes are mixed. Some researchers suggested that there is no consistent significant difference between late-preterm and full-term children from ages 4 to 15 years [8]. Other studies found that late preterm birth may be a risk factor for neurodevelopmental disorders, especially at entering school age [9–12].

The aim of this study is to explore the outcomes of neuro-motor and psycho-behavior development among LPIs aged 24~30 months. We hypothesize that, there would be significantly higher rate of motor disorders, positive M-CHAT screens and inferior cognition, among LPIs than that of full term infants.

## Methods

### Participants

There are six districts within Xi’an city of Shaanxi province, China. Two of which were randomly selected for this study comprising of 14 community health service centers. The ethics committee of Xi’an Maternal and Child Health Care Hospital approved the study and the statement of informed consent. According to the demographic data, 158 LPIs were eligible for inclusion (all of the children born between 34^+ 0^ and 36^+ 6^ weeks of gestation during Oct.1st, 2011 to Sep.30th, 2013). Out of whom, 30 LPIs’ mothers declined to participate in the study and 26 LPIs were unable to be contacted. So, only 102 parents/guardians of LPIs were interviewed on site with informed consent. One hundred fifty-three term infants (defined as 37–42 weeks’ gestational age and of birth weight between 2.5–4 kg) were randomly recruited as the control group during the same period and from the same geographical region.

### Assessment

Questionnaires regarding information about perinatal factors, and social, educational and economic states were completed by the participants’ mothers. Developmental assessment was performed by pediatric neurologists. At the time of the assessment the examiners were unaware of the fact about which of the children were born preterm. The source and references of the questionnaire are provided as an Additional file 1.

#### Chinese version of Gesell development diagnosis scale (GDDS)

There are five domains in Chinese version of the Gesell Development Diagnosis Scale (GDDS) [13] including domains of adaptability, gross motor, fine motor, language and social-emotional responses. The development quotient (DQ) of each domain were calculated for each participant. According to the full-scale DQ, the development of infants was classified as follow: normal (DQ ≥ 85), deficient (DQ < 75) and borderline (75 ≤ ~ < 85). DQ in any single domain falling below 75 was also considered as deficient within this field.

#### Modified checklist for autism (M-CHAT)

The Modified Checklist for Autism (M-CHAT) is a 23-item parent questionnaire for early identification of behaviors associated with autistic children aged 18–30 months [14]. Infants who fail 2 or more items of 2,7,9,13,14,15, or ≥ 3 items overall, are screened positive for risk of ASD or other developmental disorders. Infants positive on the screen had a follow-up check, and were assessed by a developmental pediatrician for a clinical diagnosis of autism.

#### The sensory integration schedule (SIS)

This tool uses parental observations to measure and assess children’s sensory symptoms from 0 to 12 years of age. It was revised by Dr. Zheng Xin-Xiong in Taiwan in 1985 according to the Sensory Integration Theory by Ayres, an American psychologist, but allowing for the Chinese cultural background, and is now used in China [15, 16]. It has seven sections comprising of 64 items. Raw scores are converted into standardized T-scores, which can then be used to classify the sensory integration ability as normal (T score equals 50 ± 5) and abnormal (T score < 45). In this study, only four sections were examined including vestibular balance, Cranial nerves suppression, tactile defunctness and proprioception, as these are the only sections pertinent to infants aged 24–30 months.

#### Motor delay and cerebral palsy

Infants in the study underwent a neurological examination, with cerebral palsy being diagnosed according to standard criteria [17]. Motor developmental age falling behind 3 months of the corresponding milestone or a DQ of gross motor in GDDS of < 75 can be confirmed as motor delay. Both motor delay and cerebral palsy are classified as motor disorders.

### Statistical analyses

Statistical data was processed using the Statistical Package for the Social Science (SPSS) version 18.0 for Windows. The descriptive data were presented by $\overline{x}$ ±SD. The rate of screening positive was compared between two groups using Chi-Square tests and Fisher’s exact probability. A t-test for two independent samples was used to compare the means of the groups. The association of multiple factors including birth weight, gestational age and risk factors of perinatal period to GDDS and SIS was analyzed by Person’s correlation analysis.

## Results

### The feature of the study population

Table 1 outlines the characteristic of the two groups. There was no significant difference between two groups regarding gender, birth weight (BW), delivery mode, and maternal education

### Developmental outcomes in late-preterm and full-term children at ages 24 to 30 months

**Fig. 1:**
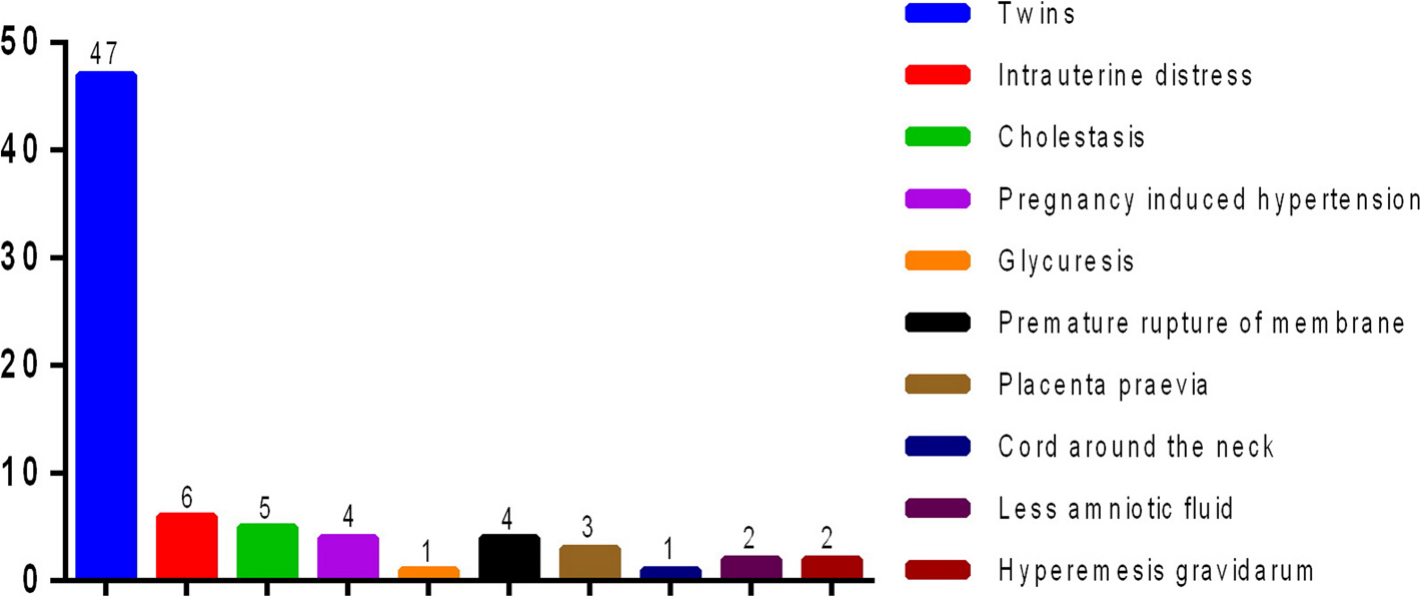
Distribution of perinatal risk factors in group 1

Comparisons of the neurodevelopment outcomes are summarized in Table 2. Two LPIs were found with CP and seven had motor delay compared to one motor delay in group 2. The rates of MD, true positive screen of M-chat, abnormity in GDDS and SIS were statistically higher than those of group 2. Furthermore, seven twins among the LPIs (7/9) were found to have positive screen of M-chat. Figure 1 shows the perinatal risk factors for developmental abnormality in LPIs.

**Table 1:** The characteristic features of the study population

	Group1	Group2	× ^2^	*P* value
	*N* = 102	*N* = 153		
Male, *n* (%)	68(66.7)	94(61.4)	0.075	0.784
GA(weeks) -mean (SD)	35.5 (1.02)	39.6(1.13)	− 22.76^a^	< 0.001
BW(gram) -mean (SD)	2796 (4820)	3465(415)	−0.996 ^a^	0.321
Deliver mode, *n* (%)			0.075	0.784
Natural labor	35(34.3)	56(36.6)		
Caesarean section	67(65.7)	97(63.4)		
Perinatal risk factors, *n* (%)	65(63.7)	0 (0)		< 0.001^b^
Maternal education, *n* (%)			< 0.001	0.986
Completion of University	59(57.8)	88(57.5)		
Completion of Secondary school	43(42.2)	65(42.5)		

^a^t-test, ^b^ Fisher’s exact probability test. Significance = < 0.05

**Table 2:** Rate of developmental abnormalities in the two groups

	Group1 *N* (%) 102	Group2 *N* (%) 153	x^2^ value	*p* value
MD	9 (8.8)	1 (0.65)		< 0.001^a^
Rate of positive screen of M-chat	9(8.8)	0 (0)		0.029 ^a^
Abnormity in GDDS	46 (45.1)	3 (5.8)	24.557	< 0.001
Abnormity in CSIC	30 (29.4)	5 (3.3)	7.686	0.006

^a^Fisher’s exact probability test

**Table 3:** Comparison of scores of GDDS between the two groups (Mean score and SD)

	Group1 *n* = 102	Group2 *n* = 153	T	*P*
Adaptability	76.91(14.42)	88.12(11.43)	−4.874	< 0.001
Gross motor	84.57(13.33)	90.59(9.85)	−2.881	0.005
Fine motor	79.23(11.90)	88.77(11.75)	−4.727	< 0.001
Language	78.72(16.43)	86.83(10.41)	−3.241	0.001
personal-social	78.91(14.34)	90.15(10.97)	−4.946	< 0.001
Vestibular balance	52.83(12.00)	61.12(9.28)	−4.356	< 0.001
Inhibition troubles of nervous system	51.14(11.54)	57.23(8.42)	−3.375	0.001
tactile defunctness	54.71(7.75)	58.75(9.71)	− 2.806	0.006
proprioception	56.06(9.28)	58.96(7.67)	−1.942	0.054

**Table 4:** Comparison of scores of SIS between the two groups (Mean score and SD)

	Group1 *n* = 102	Group2 *n* = 153	T	*P*
Vestibular balance	52.83(12.00)	61.12(9.28)	−4.356	< 0.001
Inhibition troubles of nervous system	51.14(11.54)	57.23(8.42)	−3.375	0.001
tactile defunctness	54.71(7.75)	58.75(9.71)	−2.806	0.006
proprioception	56.06(9.28)	58.96(7.67)	−1.942	0.054

**Table 5:** Distribution of scores in GDDS of LPIs with positive M-chat screening

Item	Mean Score and SD	The minimum and the peak		Distribution of scores		
			> 75	75–55	54–40	< 40
Adaptability	56.89(9.71)	[39, 70]	0	2	6	1
Gross Motor	76.11(13.88)	[44, 88]	8	0	1	0
Fine Motor	67.11(9.68)	[50, 84]	3	5	1	0
Language	56.44(10.39)	[37, 67]	0	6	2	1
personal-social	62.00(9.42)	[47, 79]	1	6	2	0

### Comparison of GDDS and the child sensory integration checklist between the two groups

Tables 3 and 4 outline the scores of GDDS and the Child Sensory Integration of the two groups. LPIs presented inferior abilities in all the regions of GDDS and much lower level in the Child Sensory Integration compared with group 2 (*p* < 0.05). Although the difference between the two groups in the category of proprioception was not statistically significant (*p* > 0.05), but LPIs still showed a low mean score in this category.

### Correlation of gestational age (GA), birth weight (BW) and the perinatal factors with various functional regions of GDDS and the child sensory integration checklist

The results of Person’s correlation analysis indicated that among perinatal risk factors, twins are associated with a statistically significant reduction in vestibular balance scores of SIS (r = − 0.203, *p* = 0.041). Pregnancy induced hypertension (PIH) is associated with adaptability and language problems (r = − 0.234, − 0.198, *p* = 0.018, 0.046), premature rupture of membrane (PROM) is associated with gross motor, fine motor and social-emotional problems according to GDDS (r = − 0.265, − 0.209, − 0.231, *p* = 0.007, 0.035, 0.020). With the increased BW and GA, scores in all the regions of GDDS, vestibular balance, Physiological inhibition of cranial nerve and SIS ascend accordingly. (r = 0.436,0.382,0.466, 0.327, 0.443, 0.249, 0.215; 0.393, 0.293, 0.406, 0.219, 0.369, 0.234, 0.260. *p* = < 0.001, < 0.001, < 0.001, < 0.001, < 0.001, 0.002, 0.008; < 0.001, < 0.001, < 0.001,0.006, < 0.001, 0.004, 0.001). The same thing can also be found between GA and tactile defunctness (r = 0.204, *p* = 0.010).

### DQ in various functional regions of GDDS for LPIs with positive screen of M-chat

LPIs that screened positive for ASD showed a disequilibrium trend in various regions of GDDS. They showed much lower DQ in adaptability, language and personal-social categories (Table 5).

## Discussion

In our study, LPIs presented negative neuropsychologic and behavior results: 8.8% of LPIs screened true positive on M-chat for ASD at 2 years, which was similar to the 4% ~ 8% for very preterm and extremely preterm infants observed in previous studies [18–20]**.** Data from the 2010 American CDC surveillance year revealed the overall prevalence of ASD reaching 14.7/1000 (one in 68 children aged 8 years) [21]**,** however risk of ASD for LPIs was 2 to 4 times greater than that for term infants. According to the reports by Guy et al. and Hwang et al., about 2.4 and 1.3%, LPIs screened true positive for ASD respectively [22, 23]. As there has been little previous investigation about the development of ASD in the LPIs population, the cause of the variation seen in positive ASD screening rates in LPIs remains unclear. The investigation methods, sample size and age of children may be factors affecting the different rates, however most importantly, many of these studies suggest a higher risk of ASD in LPIs. The etiology of ASD in late preterm twins is still poorly understood. One reason for this may be aberrant brain development playing a part in the development of ASD [18], another reason may be genetic and shared environmental factors contributing to the increased risk of ASD for twin LPIs [24–26]. Therefore, twin LPIs in particular, should receive more attention on their neuropsychological development. LPIs with positive ASD, showed unevenly distributed abilities in GDDS. Impairments in cognition greatly influence a child’s future social life and even the academic performance [27], therefore early screening for ASD and early intervention are important for these children.

Sensory Integration Dysfunction (SID) includes difficulties in receiving and processing stimuli from different senses [28]. It has been confirmed that SID is associated with white matter damage, adverse circumstance stimulation in NICU including repeated pain stimuli, and separation from parents [29]. In this study however, LPIs had none of the above factors, but they still showed problems related to vestibular balance, nervous system inhibition, and tactile defunctness, suggesting sensory modulation defect. Mitchell et al., reported the sensory modulation defect as being more common in preterm infants under 3 years [30] and our results suggested a similar conclusion, implying that LPIs may have behavioral problems in school age needing additional academic assistance **[**31**,** 32**].** The study by Olean et al., showed that 82% (59/72) of preterm infants with gestational age of < 30 weeks had abnormal sensory reactivity [33]; which our study showed to be in 29.4% of LPIs, therefore the sensory integration development of LPIs should not be neglected.

Recognition deficit is common in preterm infants and show significant correlation with low gestation and low birth weight. A study shows the “dose-response” effect of GA on later development [34]. Similar results can also be found in our study: scores in each region of GDDS were statistically lower than those of term infants. Abnormal motor development was also more common in Group 1. All of these indicate high risk of cognitive and motor developmental problems for LPIs. LPIs seemed to have a diagnosis of a development delay in the first 3 years of life with poor cognitive performance [35, 36]. Although most LPIs in this study were born without complications and had no history of neonatal diseases or experience of complex medical intervention, and seemed to show no major problems in the post-neonatal period, even then the development of recognition, motor, and social-emotion was not good as compared to term infants. A study conducted by Ozkan et al. has reported that socioeconomic risk factors were important biological risk factors in the development of children aged 3 months to 5 years [37]. Multiple factors i.e. twin pregnancy, GA, BW, PIH and PROM were found to be correlated to LPIs’ cognitive development and sensory modulation in this study. We can therefore safely conclude that an immature brain, in addition to perinatal risk factors, likely plays a crucial role in their inferior neurodevelopment. Thus LPIs, unlike term infants, shall be given more attention as preterm infants and shall be assessed and monitored for neurodevelopment impairment.

## Conclusion

In accordance with the results of the study, we have arrived at following conclusions: firstly, LPIs born with no major problems can still have increased risk of ASD, MD, SID and recognition defects. Secondly, LPIs (especially twins) may benefit from regular follow up during the first 3 years of life, for early detection of possible disorders and timely intervention could help to avoid poorer long-term outcomes. Thirdly, conservative management styles hoping for normal development can be detrimental for LPIs, therefore it is important to educate parents regarding the normal development of a child. Fourthly, some developmental domains and perinatal risk factors were identified in the study to have no significant difference; so, a longer period of follow up and a larger sample size may reveal more definitive outcomes. And finally, this study will contribute to fill the knowledge gap about the neurological and behavioral out comes of the late preterm infants, who were previously considered as normal deliveries and were not given special attention as preterm infants. So, this study clearly differentiates and emphasizes the importance of understanding about LPIs to be considered for special attention like preterm infants.

## Additional file

Additional file 1: Questionnaire sources. (DOCX 14 kb)

## Data Availability

The relevant data and materials are stored and saved with the author and the Hospital medical affairs department and is available on demand. All questionnaires used in this study are referenced both in manuscript as well as in the supplementary files.

## Abbreviations

ADHD: Attention deficit hyperactivity disorder
ASD: Autism spectrum disorders
BW: Birth weight
DQ: Development quotient
GA: Gestational age
GDDS: Gesell Development Diagnosis Scale
LPIs: Late preterm infants
M-CHAT: The modified checklist for autism in toddlers
MD: Motor disorders
SID: Sensory Integration Dysfunction
SIS: Sensory Integration Schedule
