# Supplementary material for: A study on the neurodevelopment outcomes of late preterm infants
Source: BMC Neurol. 2019 May 30;19:108. doi: 10.1186/s12883-019-1336-0 (PMC6542031; doi:10.1186/s12883-019-1336-0)
Supplement: Supplementary file 1 — Questionnaire sources. (DOCX 14 kb) [file 12883_2019_1336_MOESM1_ESM.docx]

GDDS--- Yu-feng Y.Rating Scales For Children’s Developmental Behavior and Mental Health[M]Version 1, Beijing: People's Medical Publishing House,2016:71

M-chat [Dumont-Mathieu T](https://www.ncbi.nlm.nih.gov/pubmed/?term=Dumont-Mathieu%20T%5bAuthor%5d&cauthor=true&cauthor_uid=16161090),[Fein D](https://www.ncbi.nlm.nih.gov/pubmed/?term=Fein%20D%5bAuthor%5d&cauthor=true&cauthor_uid=16161090). Screening for autism in young children: The Modified Checklist for Autism in Toddlers (M-CHAT) and other measures. [Ment Retard Dev Disabil Res Rev.](https://www.ncbi.nlm.nih.gov/pubmed/16161090)2005,11(3):253-62.

**Sensory Integration Rating Scale (SIRS)** Xinxiong Zheng. How to help children with learning difficulties[M]Version2, Beijing: Jiuzhou Publishing House,2004:251-258
